# Integrated Analysis of Microbiome and Transcriptome Data Reveals the Interplay Between Commensal Bacteria and Fibrin Degradation in Endometrial Cancer

**DOI:** 10.3389/fcimb.2021.748558

**Published:** 2021-09-21

**Authors:** Chao Li, Ye Gu, Qizhi He, Jian Huang, Yunfeng Song, Xiaoping Wan, Yiran Li

**Affiliations:** ^1^Shanghai Key Laboratory of Maternal Fetal Medicine, Shanghai First Maternity and Infant Hospital, School of Medicine, Tongji University, Shanghai, China; ^2^Department of Gynecology, Shanghai First Maternity and Infant Hospital, Tongji University School of Medicine, Shanghai, China; ^3^Department of Pathology, Shanghai First Maternity and Infant Hospital, Tongji University School of Medicine, Shanghai, China

**Keywords:** endometrial cancer, microbiome, pyrosequencing, transcriptome, biomarker

## Abstract

The gut-uterus axis plays a pivotal role in the pathogenesis of endometrial cancer (EC). However, the correlations between the endometrial microbiome and endometrial tumor transcriptome in patients with EC and the impact of the endometrial microbiota on hematological indicators have not been thoroughly clarified. In this prospective study, endometrial tissue samples collected from EC patients (n = 30) and healthy volunteers (n = 10) were subjected to 16S rRNA sequencing of the microbiome. The 30 paired tumor and adjacent nontumor endometrial tissues from the EC group were subjected to RNAseq. We found that *Pelomonas* and *Prevotella* were enriched in the EC group with a high tumor burden. By integrating the microbiome and hematological indicators, a correlation was observed between *Prevotella* and elevated serum D-dimer (DD) and fibrin degradation products (FDPs). Further transcriptome analysis identified 8 robust associations between *Prevotella* and fibrin degradation-related genes expressed within ECs. Finally, the microbial marker of *Prevotella* along with DD and FDPs showed a high potential to predict the onset of EC (AUC = 0.86). Our results suggest that the increasing abundance of *Prevotella* in endometrial tissue combined with high serum DD and FDP contents may be important factors associated with tumor burden. The microbe-associated transcripts of host tumors can partly explain how *Prevotella* promotes DD and FDPs.

## Introduction

Endometrial cancer (EC) is the most common female reproductive tract malignancy in developed countries and shows an increasing incidence (Bray et al., 2018). Importantly, the incidence of EC is drastically rising in high-income countries (Jonusiene and Sasnauskiene, 2021). Although several mechanistic events accompanying host genetic alterations and hereditary factors have been shown to play important roles in endometrial carcinogenesis, they can only explain 10–20% of cases (Walther-António et al., 2016; Kuźmycz and Stączek, 2020). Efforts to identify the cause of the remaining 80–90% of cases have led to studies on a number of environmental factors, including hormones, obesity, inflammation, and menopausal status, which are major risk factors for the development of type I EC (Beral et al., 2005; Morice et al., 2016). There is an urgent need to identify previously unrecognized mechanisms for the diagnosis and therapeutic management of EC. However, the underlying mechanisms involved in the occurrence and development of EC are far from being explored.

Available evidence has shown the potential involvement of microbiota in the development of different types of human cancer, including endometrial cancer (Lu et al., 2021). Of interest, several cross-sectional studies have noted a link between microbiota composition and EC (Walther-António et al., 2016; Walsh et al., 2019; Lu et al., 2021). Therefore, endometrial microbiota could be implicated as an environmental influence that contributes to the progression of EC. Nonetheless, the profile of the endometrial microbial community and its function in endometrial carcinogenesis remain unclear. Only one study has explored whether the increased abundance of *Micrococcus* is positively correlated with IL-6 and IL-17, which are involved in the proinflammatory response in EC (Lu et al., 2021). Host genes are known as important regulators connecting microbiota to cancer (Huang et al., 2020). Microbiota can alter the expression of host genes and exert a tumor stimulative role through multiple mechanisms, such as by modifying signaling proteins and modulating subsequent transcriptional responses (O’Keefe, 2016). However, the interplay between the endometrial microbial community and host transcriptome and their roles in EC development have not been addressed.

In this prospective study, we investigated the endometrial microbiota composition in a cohort of 10 healthy controls (HCs) and 30 EC patients using high pyrosequencing of barcoded 16S rRNA genes (V3–V4). The transcriptome of the paired tumor and adjacent nontumor endometrial tissues from the 30 EC patients was also determined. By the simultaneous integrated analysis of the endometrial tumor transcriptome, endometrial microbiome, and hematological indicators, we aimed to gain better insights into the nature of the gut-transcriptome-uterus axis in EC patients.

## Methods

### Participant Information

This study was conducted at Shanghai First Maternity and Infant Hospital affiliated with Tongji University from March 2018 to July 2020. The inclusion criteria of the participants were as follows: (a) women aged between 40 and 69 years; and (b) subjects undergoing hysterectomy by any standard surgical approach for any possible benign disease and at stage I endometrial cancer (EC). The exclusion criteria were as follows: (a) pregnant women and nursing mothers; (b) women who used antibiotics within 3 months; (c) history of genital tract infection or medication within 3 months; and (d) patients receiving preoperative chemotherapy or radiotherapy. A total of approximately 120 subjects were recruited, and after filtering with the inclusion and exclusion criteria, 40 women, including 30 endometrial cancer (EC) patients and 10 healthy controls (HCs), were finally enrolled (**Table S1**).

Endometrial tissue samples were acquired from these 40 women, who had undergone a hysterectomy. After removing the corrupted and erosive tissues of the surface, the residual tissues were macrodissected. The whole process was performed by an experienced gynecologist under strict aseptic procedures. The samples were divided into three equal parts (one for 16S rRNA sequencing, one for RNA-seq, and one for subsequent experimental verification), snap frozen and stored at −80°C.

### Bacterial DNA Extraction and Amplification

Bacterial DNA was isolated from the endometrial samples using a DNeasy PowerSoil kit (Qiagen, Hilden, Germany) following the manufacturer’s instructions. DNA concentration and integrity were measured by a NanoDrop 2000 spectrophotometer (Thermo Fisher Scientific, Waltham, MA, USA) and agarose gel electrophoresis, respectively. PCR amplification of the V3-V4 hypervariable regions of the bacterial 16S rRNA gene was carried out in a 25 μL reaction using universal primer pairs (343F: 5′-TACGGRAGGCAGCAG-3′; 798R: 5′-AGGGTATCTAATCCT-3′) (Chen et al., 2018). The reverse primer contained a sample barcode, and both primers were connected with an Illumina sequencing adapter.

### Library Construction and Sequencing

The amplicon quality was visualized using gel electrophoresis. The PCR products were purified with Agencourt AMPure XP beads (Beckman Coulter Co., USA) and quantified using a Qubit dsDNA assay kit. The concentrations were then adjusted for sequencing. Sequencing was performed on an Illumina MiSeq with two paired-end read cycles of 300 bases each (Illumina Inc., San Diego, CA; OE Biotech Company; Shanghai, China). The raw data for the Illumina reads from 40 endometrial samples were uploaded to the Sequence Read Archive (SRA) database.

### Bioinformatic Analysis

Paired-end raw sequences were preprocessed using Trimmomatic software (version 0.36) (Bolger et al., 2014) to detect and cut off ambiguous bases (N), and this software was also used to cut off low-quality sequences, with an average quality score below 20 using a sliding window trimming approach. After trimming, paired-end reads were assembled using FLASH software (version 1.2.11) (Reyon et al., 2012). Sequences were further denoised using QIIME software (version 1.8.0) (Caporaso et al., 2010). Then, reads with chimeras were detected and removed using VSEARCH (version 2.4.2) (Rognes et al., 2016). Clean reads were subjected to primer sequence removal and clustering to generate operational taxonomic units (OTUs) using VSEARCH software, with a 97% similarity cutoff (Rognes et al., 2016). The representative read of each OTU was selected using the QIIME package. All representative reads were annotated and blasted against the Silva database (Version 123) using the RDP classifier (confidence threshold was 70%) (Wang et al., 2007). The microbial diversity in the endometrial samples was estimated using the alpha diversity, which includes the Chao1 index, observed species index, Simpson index, and Shannon index (Yang et al., 2019). The unweighted UniFrac distance matrix performed by QIIME software (version 1.8.0) was used for nonmetric multidimensional scaling (NMDS) plots. A Venn diagram was generated to visualize the shared and unique genera among groups regardless of their relative abundance using the R package “VennDiagram” (Zaura et al., 2009). The LEfSe (linear discriminant analysis effect size) method was used to detect differentially abundant taxa across groups using the default parameters (Segata et al., 2011). 16S rRNA gene amplicon sequencing and analysis were conducted by OE Biotech Co., Ltd. (Shanghai, China).

### RNA Extraction, RNA-Seq, and Data Analysis

Total RNA was extracted from frozen endometrial tissues by using mirVana™ miRNA Isolation Kit (catalog no., AM1561, Ambion^®^), and cDNA libraries were sequenced on the MGISeq2000 Platform (MGI, Shenzhen, China). Then, 150 bp paired-end reads were generated. Approximately 49.35 million raw reads for each sample were generated. Raw data (raw reads) in fastq format were first processed using Trimmomatic software (Bolger et al., 2014), and the low-quality reads were removed to obtain clean reads. Then, approximately 48.57 million clean reads for each sample were retained for subsequent analyses.

The clean reads were mapped to the human genome (GRCh38) using HISAT2 (version 2.2.1.0) (Kim et al., 2015). The FPKM (Roberts et al., 2011) value for each gene was calculated using Cufflinks (version 2.2.1) (Trapnell et al., 2010), and the read counts of each gene were obtained by HTSeq-count (version 0.9.1) (Anders et al., 2015). A differential expression analysis was performed using the DESeq (2012) R package (version 3.2.0, R Foundation for Statistical Computing, Vienna, Austria). A p value < 0.05 and fold change > 2 or fold change < 0.5 were set as the thresholds for significantly differential expression. A hierarchical cluster analysis of differentially expressed genes (DEGs) was performed to demonstrate the expression pattern of genes in different groups and samples. GO enrichment and KEGG pathway enrichment analyses of DEGs were performed using R based on the hypergeometric distribution.

### Correlation Between the Genus and Clinical Hematological Characteristics

The Pearson correlation coefficient was calculated to evaluate the clinical characteristics that underwent significant changes (values of serum D-dimer (DD), fibrin degradation products (FDPs), cancer antigen (CA) 125, CA199, and total protein) and endometrial microbiota at the genus level between HC and EC women. To reduce the computational load and avoid contingency, the genera that presented an abundance value of ≤ 0.1% were excluded. The significance of each genus-hematological indicator pair was determined based on a *p* value < 0.05 and a false discovery rate (FDR) < 0.1. Detailed scripts of the correlation calculations are provided in **Table S2**.

### Correlation Between Genera and Differentially Expressed Genes

The Pearson correlation coefficient was calculated to measure the connections between genus abundance and differential gene expression level for each genus-gene pair across all 30 patients. To reduce the computational load and avoid contingency, the genera that presented an abundance value of ≤ 0.1% were excluded. The significance of each genus-gene pair was determined based on a *p* value < 0.05 and FDR < 0.1. Independent Student’s *t* test was applied to evaluate the difference in log_2_FC values calculated by DESeq between the HC and EC groups. Detailed scripts of the correlation calculations are provided in **Table S3**.

### Immunofluorescence Staining

Paraffin-embedded endometrial tissue sections were treated as previously described (Li et al., 2019). After retrieval of antigenic binding sites, the sections were permeabilized with 0.2% Triton X-100 for 10 min, and then blocked with 5% bovine serum albumin for up to 1.5 h at room temperature, and incubated with appropriate concentrations of primary antibodies against PRSS33 (Abmart, Shanghai, China, TD4461M, 1:200 dilution), CPB2 (Bioss Antibodies, Beijing, China, BS-7547R, 1:200 dilution), and XBP1 (Abcam, London, UK, ab37152, 20 µg/mL) overnight at 4°C. Then, the sections were incubated with Alexa Fluor^®^ 488/594/647 conjugated goat anti-rabbit IgG (Abcam, London, UK, 1:200 dilution) secondary antibodies for 1 h. Cell nuclei was stained with DAPI. The stained sections were observed under a fluorescence confocal microscope.

### Statistical Analysis

Fundamental statistical analyses were performed using SPSS software (version 19.0, IBM SPSS Inc., Chicago, IL, USA), GraphPad Prism 7 software (GraphPad software, Inc., San Diego, California, USA) and R version 3.2.0. Categorical variables were expressed as numbers (%). For normally distributed variables, the Student’s *t* test was used; otherwise, the Wilcoxon rank sum test was used. Pearson’s rank correlation analysis was conducted to calculate the correlation between genera, between genera and clinical characteristics, or between genera and DEGs. All statistical tests were two-tailed, and the *p* value was adjusted by the Benjamini-Hochberg correction. A *p* value < 0.05 was considered statistically significant.

## Results

### Summary of Clinical Characteristics

All 30 endometrial cancer (EC) patients and 10 healthy control (HC) women were Han Chinese individuals from Shanghai. All diagnoses were performed based on the final surgical pathology. The demographics and other clinicopathological characteristics are shown in **Table 1**. The control participants were generally matched with the case participants by age and BMI, suggesting that there were no established confounding factors that might influence group discrimination prior to sample collection. There was a significant increase in serum DD (*p* = 0.0122), FDP (*p* = 0.0003), CA125 (*p* = 0.0094) and CA199 (*p* = 0.0261) levels and a significant decrease in serum total protein in EC patients (*p* = 0.0441) (**Table S1**).

**Table 1:** Clinical characteristics summary of all enrolled women.

Category	Healthy control(n = 10)	Endometrial cancer group(n = 30)	*P* value
*Clinical and pathological feature*			
Age (year)	53.1 ± 6.67^a^	56.4 ± 7.89^a^	0.1042
Body mass index (kg/m^2^)	22.89 ± 2.15^a^	22.70 ± 1.66^a^	0.4072
Histology			–
Endometrioid	–	22 (73.3%)^b^	
Serous	–	8 (26.7%)^b^	
FIGO stage			–
I-II	–	30 (100%)^b^	
III-IV	–	0 (0%)^b^	
*Hematologic biomarker* ^a^			
Total protein (64.0-83.0 g/L)	74.49 ± 1.73	72.42 ± 5.76	0.0441
Albumin (35.0-55.0 g/L)	45.86 ± 2.32	44.73 ± 4.02	0.1422
Globulin (20.0-35.0 g/L)	28.63 ± 2.30	27.69 ± 3.72	0.1771
Prothrombin time (11-13 s)	11.17 ± 0.74	11.0 ± 0.59	0.26
Fibrinogen (2.0-4.0 g/L)	2.43 ± 0.48	2.65 ± 0.61	0.13
D-dimer (0-0.5 mg/L)	0.19 ± 0.07	0.42 ± 0.52	0.0122
Fibrin degradation products (0-5.0 mg/L)	0.96 ± 0.45	1.91 ± 1.70	0.0045
Cancer antigen 125 (0-35.0 U/mL)	8.20 ± 5.34	29.37 ± 45.87	0.0094
Cancer antigen 199 (0-37.0 U/mL)	5.17 ± 2.79	46.43 ± 111.49	0.0261
Alpha fetoprotein (0-20.0 mg/L)	2.63 ± 0.94	3.13 ± 1.30	0.1008
Carcinoembryonic antigen (0-5.0 mg/L)	1.64 ± 0.82	1.93 ± 1.36	0.2138

a Values are given as mean ± SD.

b Categorical variables were expressed as numbers (%).

–, not exist.

### Decreased Bacterial Diversity in Endometrial Microbiota Associated With EC

**Figure 1:**
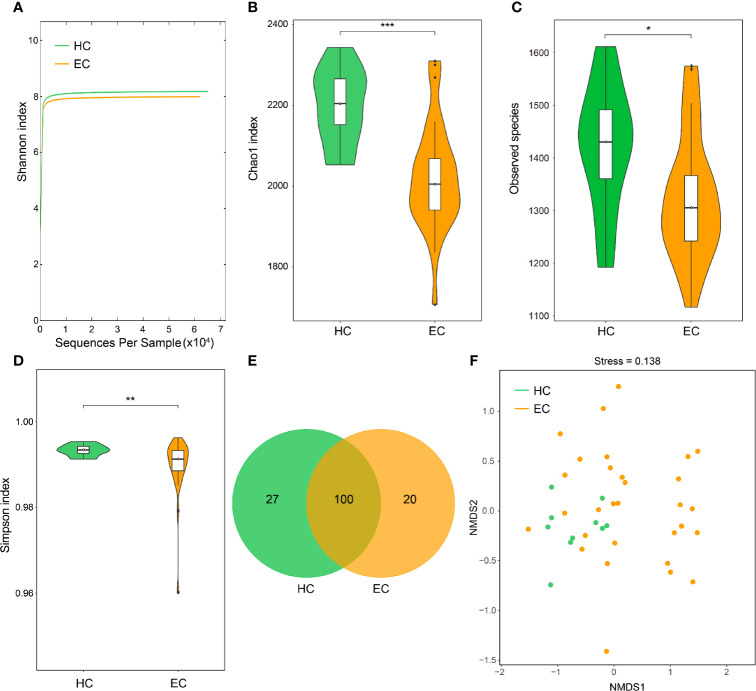
Endometrial microbial diversity of HC and EC participants. **(A)** Shannon-Wiener rarefaction curve of HC and EC groups. **(B–D)** α-Diversity comparison between different disease states in the endometrial microbiome. **(B)**, Chao1 index; **(C)**, observed species; and **(D)**, Simpson index. ^*^ *p* < 0.05, ^**^ *p* < 0.01, ^***^ *p* < 0.001, Wilcoxon rank sum test. **(E)** Venn diagram illustrating the shared and unique genera between HC and EC. **(F)** Beta diversity evaluated using NMDS with an unweighted UniFrac distance matrix. HC, healthy control; EC, endometrial cancer.

We analyzed a total of 40 endometrial samples based on the pyrosequencing of barcoded 16S rRNA genes (V3–V4) and finally obtained 2,728,218 qualified sequences (median =  68,151) and 9,278 OTUs (**Tables S4**, **S5**). The rarefaction curves indicated that the estimated richness of OTUs almost reached saturation in the HC (n = 10) and EC groups (n = 30) (**Figure 1A**). Moreover, the rank abundance curves indicated decreased richness in the EC patients compared with the HCs (**Figure S1**).

To estimate the differences in bacterial diversity between the two groups, the sequences were aligned to estimate alpha diversity and beta diversity (**Table S6**). Significant differences between the EC and HC groups were observed in the Chao1 (2247.72 ± 151.01 *versus* 2476.16 ± 142.47, *p* < 0.001), observed species (1321.61 ± 114.95 *versus* 1420.98 ± 117.80, *p* = 0.0212) and Simpson (0.989 ± 0.007 *versus* 0.993 ± 0.001, *p* = 0.0053) indexes (**Figures 1B–D**), whereas significant differences between these groups were not observed for the Shannon index (7.99 ± 0.41 *versus* 8.18 ± 0.26, *p* = 0.0606) (**Figure S2**). A Venn diagram showed that 100 out of 147 genera were shared between the EC and HC groups (**Figure 1E**). Notably, 20 out of 147 genera were unique to the EC group. For the beta diversity, the NMDS plots evaluated by the unweighted UniFrac distance matrix exhibited a separation of the distribution of the two groups (**Figure 1F**). The results suggest that the diversity of endometrial microbiota could be strongly influenced by the tumor burden.

### Alterations in the Composition of Endometrial Microflora Associated With EC

**Figure 2:**
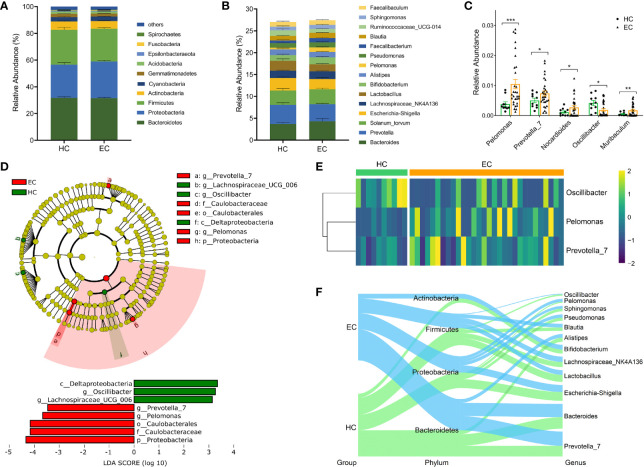
Phylogenetic profiles of endometrial microbiota between the HC and EC groups. **(A)** Compositions of the bacterial community at the phylum level between HC and EC. **(B)** Compositions of the bacterial community (top 15) at the genus level between HC and EC. **(C)** Differential microbial community at the genus level in EC *versus* HC. Error bars are presented as the SD. **(D)** LEfSe analysis for comparing microbial variations in HC and EC at the genus level. LEfSe cladogram representing differentially abundant taxa (*p* < 0.05); LDA scores are calculated based on the LEfSe of differentially abundant taxa among groups, and only taxa with LDA scores of > 3 are presented. **(E)** Distributions of *Oscillibacter*, *Pelomonas*, and *Prevotella* normalized by a *Z*-score between HC and EC. **(F)** Sankey analysis of HC and EC. *p < 0.05, **p < 0.01, ***p < 0.001.

The bacterial distribution was assessed according to the relative abundance of different taxa. The five dominant phyla in the EC and HC groups were Bacteroidetes, Proteobacteria, Firmicutes, Actinobacteria, and Cyanobacteria, which accounted for 92.17% and 92.67% of the total OTUs, respectively (**Figure 2A**). The compositions of the bacterial community (top 15) at the genus level are shown in **Figure 2B**. To provide insight into the differences in endometrial microflora between the two groups, the Wilcoxon rank sum test was performed at both the phylum and genus levels (**Tables S7**, **S8**). Compared with the HC group, *Pelomonas*, *Prevotella*, *Nocardioides* and *Muribaculum* were enriched in the EC group, whereas *Oscillibacter* exhibited the opposite trend (all *p* < 0.05, **Figure 2C**).

Next, the LEfSe method was used to discover high-dimensional biomarkers. Of the above five genera, *Pelomonas* and *Prevotella* were significantly overrepresented [LDA scores (log_10_) > 3] in the endometrium of EC patients (**Figure 2D**). The relative abundances of the two genera were further subjected to a cluster analysis, and the results suggested that *Pelomonas* and *Prevotella* were abundant in the EC group (**Figure 2E**). Sankey diagrams were also generated to visualize the major proportion of taxa (phylum and genus) between the HC and EC groups. In patients with a tumor burden, the proportion of *Pelomonas* and *Prevotella* gradually increased and became predominant, accompanied by changes in other bacteria (**Figure 2F**). Overall, these data suggest that the differentially abundant microbiota, including *Pelomonas* and *Prevotella*, were able to distinguish the microbiota between the HC volunteers and EC patients.

### Serum DD and FDPs Were Associated With Altered Microflora in EC

**Figure 3:**
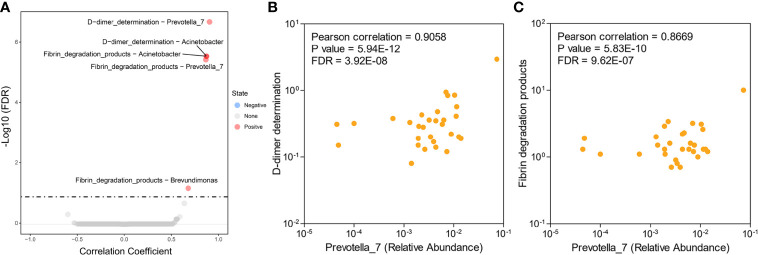
Associations between endometrial microbiota and hematological indicators in patients with EC. **(A)** Differential level of the microbe-associated hematological indicator from 30 EC samples. **(B, C)** Scatter plots of two *Prevotella*-related hematological indicators: *Prevotella*-D-dimer **(B)** and *Prevotella*-fibrin degradation products **(C)**. Each point represents a patient.

For the intraindividual correlation analyses, we tested the associations between clinical characteristics (abnormal values of serum DD, FDPs, CA125, CA199, and total protein) and endometrial microbiota. Pearson’s correlation-based analysis indicated that the three genera *Prevotella*, *Acinetobacter* and *Brevundimonas* were remarkably associated with fibrin degradation (DD and FDPs), while meaningful results based on other clinical characteristics were not observed (**Figure 3A**, **Table S2**). Among the three genera, *Prevotella* microbes were correlated with the aforementioned different endometrial microbiota composition alterations in the HC and EC groups (**Figures 3B, C**). Therefore, *Prevotella* were selected for further study.

### Global Overview of Endometrial Tumor Transcriptome in EC

**Figure 4:**
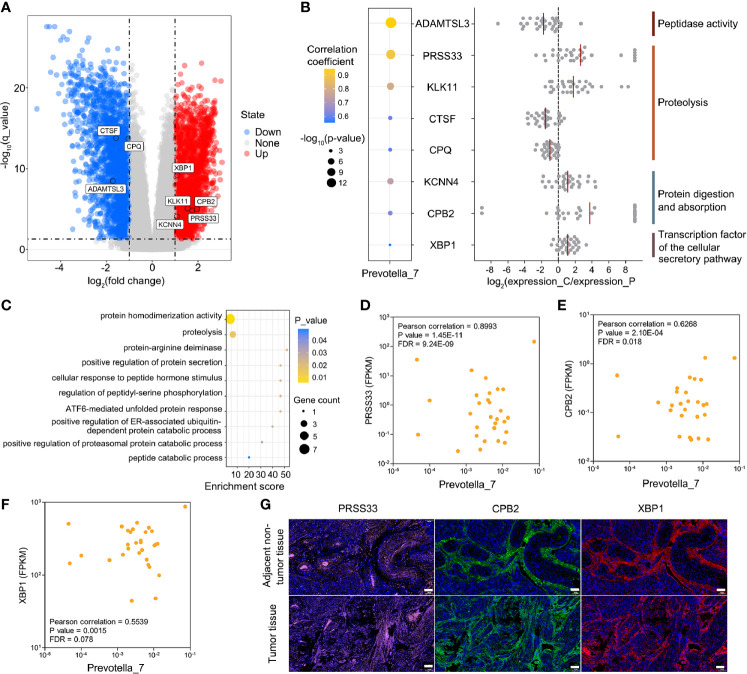
Associations between host endometrial gene expression and *Prevotella* in patients with EC. **(A)** Differential expression of *Prevotella*-associated genes from 30 paired tumor and adjacent nontumor endometrial tissue samples. **(B)** Integrated analysis of *Prevotella*-associated genes: microbe-gene correlation dot plot (left panel), log_2_FC value of genes from each patient (middle panel), and functional annotation (right panel). The red line represents the average value. **(C)** GO enrichment analysis based on the Metascape platform. **(D–F)** Scatter plots of three typical *Prevotella*-gene pairs: *Prevotella*-PRSS33 **(D)**, *Prevotella*-CPB2 **(E)** and *Prevotella*-XBP1 **(F)**. Each point represents a patient. **(G)** Immunofluorescence showed the expression levels of PRSS33, CPB2 and XBP1 in endometrial tumor and adjacent non-tumor tissues. The white scale bars are 100 μm.

Since the EC patients demonstrated signature microbiota associated with clinical characteristics (tumor burden), we hypothesized that changes in the transcriptome of endometrial tumorigenesis may be correlated with endometrial microbiota. Thus, we performed a transcriptome analysis of the paired tumor and adjacent nontumor endometrial tissues from 30 EC patients. Based on our definition, we identified a total of 3,911 differentially expressed genes (with 1,852 upregulated and 2,059 downregulated) among the 30 paired endometrial tissues by DESeq (**Figure 4A**). The genes whose log_2_FC values calculated by GFOLD were zero in > 90% of patients were excluded, thus leaving 3,410 genes for further investigation of the correlations with *Prevotella*.

### *Prevotella* Promotes DD and FDPs by Influencing the Host Endometrial Transcriptome Profile

Furthermore, Pearson’s correlation-based analysis was performed to discover *Prevotella*-associated genes and to examine whether the levels of DD and FDPs could be partially influenced by these genes. Among the 3410 genes, 74 genus-gene pairs were formed with FDR < 0.1 (**Table S3**). A total of 8 genes were identified to potentially affect the degradation of fibrin (peptidase activity, proteolysis, protein digestion and absorption, and cellular secretory pathway transcription factor) (**Figure 4B**). The pathway analysis confirmed that these 8 genes converged mainly on proteolysis-related pathways (**Figure 4C**). A subsequent correlation analysis showed three examples of *Prevotella*-gene pairs (*Prevotella*-serine protease 33 (PRSS33), *Prevotella*-carboxypeptidase B2 (CPB2), *Prevotella*-x-box binding protein 1 (XBP1)) (**Figures 4D–F**). The mammalian PRSS33 is primarily responsible for degrading fibrin into DD and FDPs within blood (Izem et al., 2021). CPB2 is involved in the clotting-fibrinolytic pathway, and abnormal expression of this protein has been linked to thromboembolism and cancer (Lip et al., 2002). XBP1 is a key transcription factor of the cellular secretory system, and it is often overexpressed in cancers, such as oral squamous cell carcinoma and hepatocellular carcinoma, and correlates with the clinical outcome (Stelloo et al., 2020). Multiplex immunofluorescence confirmed that PRSS33, CPB2 and XBP1 proteins were significantly highly expressed in the endometrial tumor tissues of patients with elevated DD and FDPs (**Figure 4G**), suggesting that *Prevotella* could partially promote DD and FDPs by influencing host gene expression. To further validate the other 2 genes (KCNN4 and KLK11) under tumor burdens, DESeq was used to calculate the correlation between *Prevotella* and the KCNN4 and KLK11 genes (**Figure S3**). The results showed that these two genes satisfied the definition of differential genes between EC and adjacent nontumor endometrial tissues.

### Identification of Microbial Blood-Based Markers for Clinical Prediction

**Figure 5:**
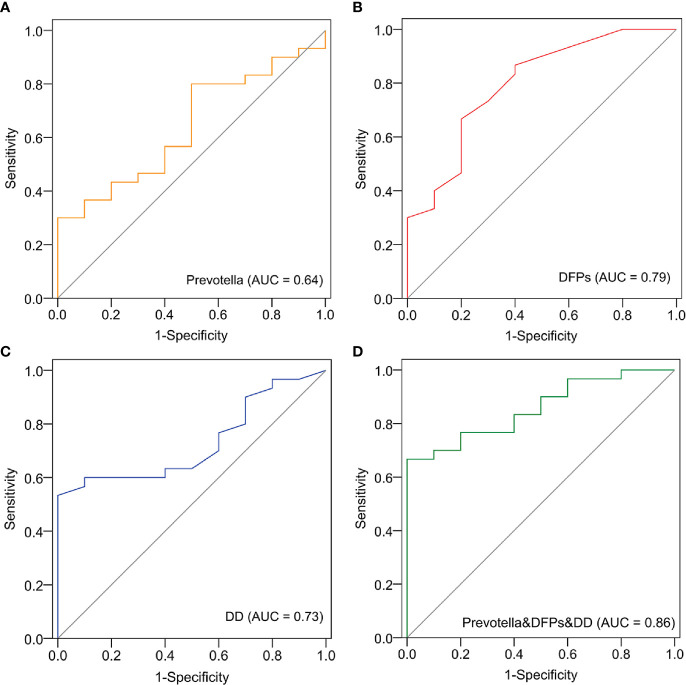
Identification of *Prevotella*-based markers for the clinical prediction of EC. The receiver operating characteristic (ROC) curve and area under the curve (AUC) were generated to predict the onset of EC based on *Prevotella* **(A)**, fibrin degradation products (DFPs) **(B)**, D-dimer (DD) **(C)**, and joint modeling of the three factors **(D)**.

According to the abovementioned analysis, we focused on *Prevotella*, DD and FDPs as potential biomarkers because they were abundant in the EC group. A receiver operating characteristic (ROC) curve analysis indicated that *Prevotella* (area under the curve (AUC) = 0.64; *p* = 0.047), DD (area under the curve (AUC) = 0.73; *p* = 0.031), and FDPs (AUC = 0.79; *p* = 0.005) were significantly associated with EC samples (**Figures 5A–C**). In addition, a ROC model in combination with the three could increase the AUC value to 0.86 (*p* = 0.001) (**Figure 5D**). The data indicated that these three biomarkers related to fibrin degradation have the potential to predict EC occurrence.

## Discussion

Symptoms of EC, such as abnormal vaginal bleeding, usually present quickly after cancer onset, and most EC patients are diagnosed at an early stage. To elucidate an early disease role for these specific microbes, our study selected women with grade I EC. We provided evidence that the tumor burden is associated with the presence of specific endometrial microbiota, which is distinguished by the enrichment of *Pelomonas* and *Prevotella* in patients with EC. Of the two microbes, *Prevotella* was found to be positively correlated with the increased serum DD and FDPs in the EC group. Further analyses of the host endometrial transcriptome revealed that *Prevotella* promotes DD and FDPs by influencing the expression of genes related to fibrin degradation. Thus, the results herein on endometrial samples shed light on the gut-transcriptome-uterus axis and may allow for the identification of EC-related biomarkers for clinical prediction.

Compared with the HC group, a significant reduction in alpha diversity was observed in the EC group. Reduced microbial diversity has been reported to lead not only to the outgrowth of a few species and decreased microbial resilience, which is prejudicial to health, but also to chronic diseases, such as cancer (Richard et al., 2018; Liu et al., 2019; Lu et al., 2021). To date, several studies have explored the correlation of microbiota diversity and endometrial disease severity. In one study, Walther-António et al. showed that alpha diversity was increased in the EC group (Walther-António et al., 2016). Other evidence has shown that advanced disease severity is associated with decreased endometrial microbiota diversity (Walsh et al., 2019; Lu et al., 2021). The differences could also be attributed to other factors, such as the geographic area, 16S rRNA gene target region, and sequencing method (Huang et al., 2018). Nevertheless, our results support the latter point of view.

Compared with previous studies that identified *Micrococcus* (Lu et al., 2021), *Atopobium vaginae* and *Porphyromonas* (Walther-António et al., 2016), or *Porphyromas somerae* (Walsh et al., 2019) as predictive microbial markers of EC, our results suggest that a higher abundance of *Prevotella* might favor the development of endometrial tumorigenesis, especially when combined with high serum DD and FDPs. *Prevotella*, a gram-negative anaerobic bacterial genus, is part of the normal flora within the mouth and vagina (Precup and Vodnar, 2019). Recent studies have suggested a potential role of *Prevotella* as an intestinal pathobiont related to oral cancer (Karpiński, 2019), colorectal cancer (Flemer et al., 2017; Peng et al., 2020), and lung cancer (Tsay et al., 2018). In particular, this genus has been recognized as a prominent gynecologic and obstetric pathogen positively associated with cervical lesions, including bacterial vaginosis (Marconi et al., 2012), cervical intraepithelial neoplasia (Mitra and MacIntyre, 2020), cervical cancer (Kang et al., 2020), and intrauterine infections (Knoester et al., 2011). Herein, it is interesting to note that *Prevotella* has an important role in the metabolism of proteins and peptides because many of the members within this genus are actively proteolytic and possess characteristic dipeptidyl peptidase activity (Flint and H., 2014). Based on the correlation of *Prevotella* with the disease along with its association with the degradation of DD and FDPs, this genus may be involved in the etiology of conditions leading up to EC.

Our results explained the internal reasons for the elevation of DD from the perspective of endometrial microbiota. DD is an end degradation product of fibrin (Kogan et al., 2016), that is considered a diagnostic and prognostic parameter in several malignancies (Fregoni et al., 2012; Ma et al., 2014; İnal et al., 2015). For example, DD was proven to be directly and positively correlated with EC and its prognosis (Nakamura et al., 2016; Ge et al., 2020). Consistent with previous findings, an elevated DD level was also observed in our study. However, the causes of such an increase are still unclear. Intriguingly, after performing transcriptome analyses, we found altered expression of genes related to fibrin degradation in endometrial tumor tissues. Further correlation-based analysis revealed that these genes were attributed to the increased abundance of *Prevotella*, suggesting that *Prevotella* might be an important factor that mediates host fibrin degradation.

Vein thrombosis is a common complication in the natural history of malignancies, and patients with cancer have a 4- to 6-fold increased risk of developing vein thrombosis compared to patients without cancer (Di Nisio et al., 2010; MacLellan et al., 2012). DD is a signal of the activated coagulation system, and it is currently used as an indicator to assess vein thrombosis probability (Kogan et al., 2016; Khan et al., 2021). Many studies have evaluated the relationship between elevated DD levels and vein thrombosis risk and found that patients with pancreatic, bladder, ovarian and endometrial cancer have the highest incidence of vein thrombosis (Peng et al., 2021). In the present study, we show that *Prevotella* increased the expression of fibrin degradation-related genes and promoted their protein levels in the EC group, suggesting that *Prevotella* could be a potential participant in venous thrombosis. Thus, to reduce vein thrombosis in clinical EC, the balance between *Prevotella* and other microbes should be maintained.

Notably, PRSS33 and XBP1 might be associated with fibrin degradation. Degradation of fibrin is attributed to plasmin, which is one of the most potent serine proteases found in many physiological processes, such as thrombolysis and cancer progression (Deryugina and Quigley, 2012). In our study, the expression of PRSS33 was significantly increased in endometrial tumor tissues, suggesting the functional effect of this protein on fibrin degradation. Although the fibrinolytic activity of PRSS33 was not verified, many studies have described the putative role of serine proteases in hemostasis/thrombosis because plasmin is a nonspecific proteolytic enzyme (Kido et al., 1999; Uesugi et al., 2011; van der Plas et al., 2014; Kakizoe et al., 2016). XBP1 is a transcription factor of the cellular secretory pathway that has been found to be upregulated in hepatic artery thrombosis (Nemes, 2008). Interestingly, a recent study indicated that the overaccumulation of PRSS was accompanied by upregulated XBP1 in a mouse model of chronic pancreatitis. Given the correlation for PRSS33 and XBP1, it is reasonable to speculate that the two proteins may be involved in the elevated DD levels.

Our study has some limitations. First, this is a cross-sectional retrospective study without follow-up data, such as overall survival (OS) and disease-free survival (DFS) data. Hence, we could not determine the classification performance of the *Prevotella*-fibrin degradation-related genes for discriminating OS or DFS. Second, information about the incidence of venous thrombosis is not provided, and further study regarding this point is suggested. Third, our sample size was relatively small. However, we were still able to identify significant microbiota differences between HCs and ECs and identify specific altered genes that are positively associated with *Prevotella* and serum DD. Nevertheless, further investigations with an expanded sample size and a multicenter survey are needed to validate the effect of the reported findings.

In summary, we have provided a distinct microbiome signature in patients with EC and healthy controls. The identified *Prevotella* in the endometrium is associated with the presence of EC, especially when combined with a high concentration of serum DD and FDPs. *Prevotella*-associated transcripts of host tumors can partly explain how endometrial microbiota promote EC pathogenesis and lead to these aberrant hematologic biomarkers. These findings provide new insights into the connections between the endometrial microbiome-transcriptome and biomarker development in the early detection of EC.

## Data Availability Statement

All raw sequences for 16S rRNA sequencing were deposited in the NCBI Sequence Read Archive under accession number PRJNA750303. All RNA-seq data generated during this study are available at NCBI GEO under accession number GSE183185. All datasets generated for this study are included in the article/**Supplementary Material**.

## Ethics Statement

This research was approved by the Scientific and Ethical Committee of the Shanghai First Maternity and Infant Hospital affiliated with Tongji University, which is accredited by the National Council on Ethics in Human and Animal Research (KS2033). All human tumor tissues were obtained with written informed consent from patients prior to participation in the study.

## Author Contributions

XW and YL designed research. CL, YG, QH, JH, YS, and YL analyzed data. CL and YG wrote the paper. CL, XW, and YL revised the paper. All authors contributed to the article and approved the submitted version.

## Funding

This work was supported by the National Natural Science Foundation of China (32070583) and Shanghai Municipal Health Commission (20194Y0156).

## Conflict of Interest

The authors declare that the research was conducted in the absence of any commercial or financial relationships that could be construed as a potential conflict of interest.

## Publisher’s Note

All claims expressed in this article are solely those of the authors and do not necessarily represent those of their affiliated organizations, or those of the publisher, the editors and the reviewers. Any product that may be evaluated in this article, or claim that may be made by its manufacturer, is not guaranteed or endorsed by the publisher.
