# Supplementary material for: Integrated Analysis of Microbiome and Transcriptome Data Reveals the Interplay Between Commensal Bacteria and Fibrin Degradation in Endometrial Cancer
Source: Front Cell Infect Microbiol. 2021 Sep 21;11:748558. doi: 10.3389/fcimb.2021.748558 (PMC8490766; doi:10.3389/fcimb.2021.748558)
Supplement: Supplementary file 1 [file Table_1.docx]

Supplementary Material

# Supplementary Figures


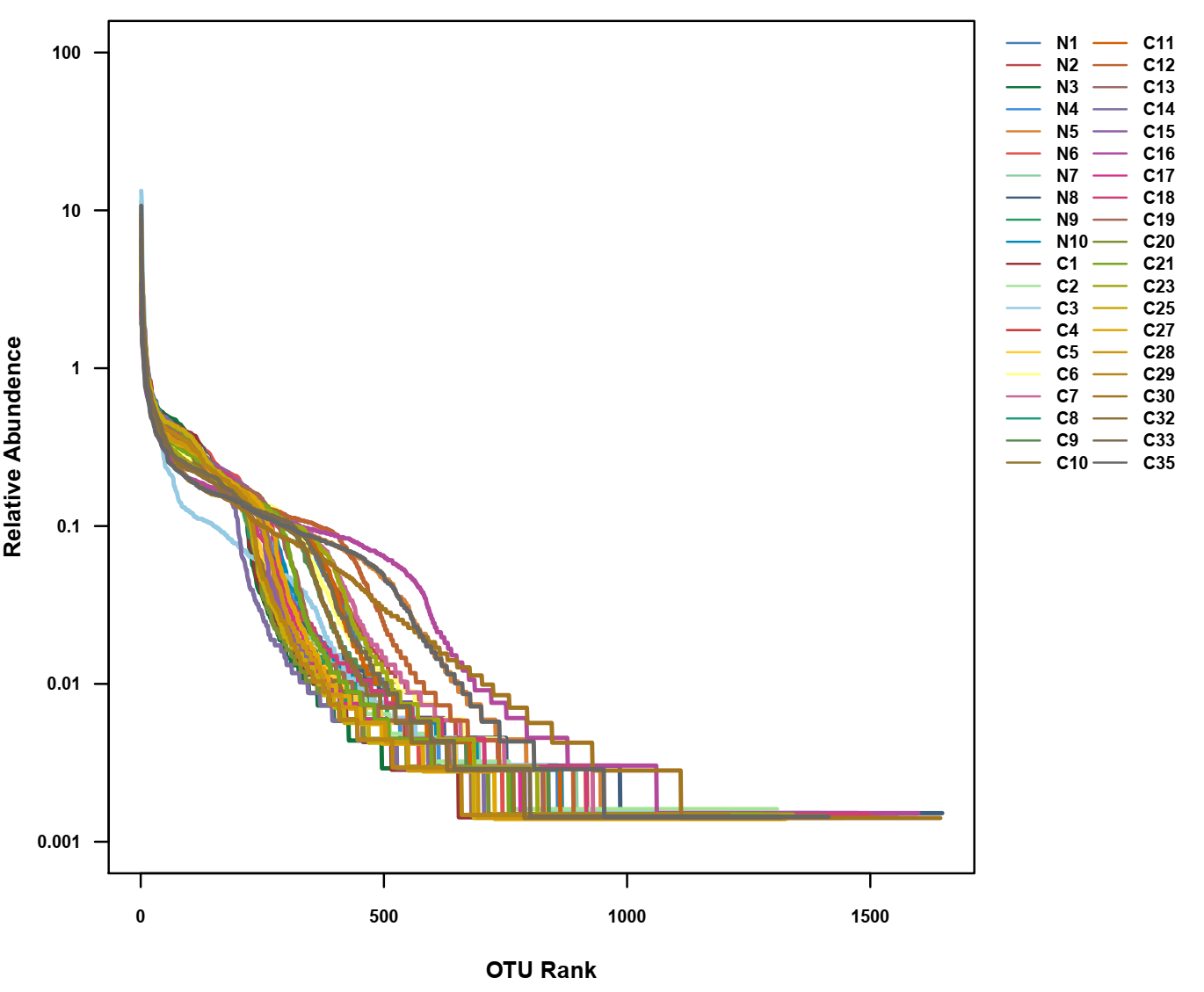


**Supplementary Figure 1.** Rank abundance curves for all OTUs.


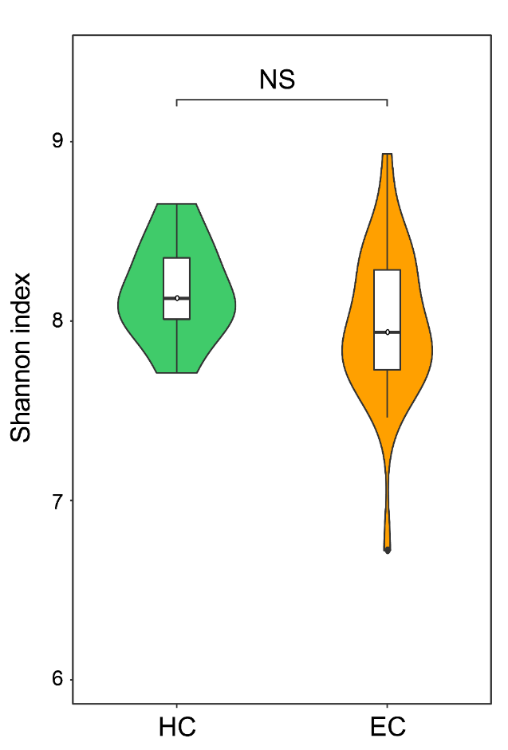


**Supplementary Figure 2.** α-diversity (Shannon index) comparison between different disease states in the endometrial microbiome. ^*^*p* < 0.05, Wilcoxon rank sum test.


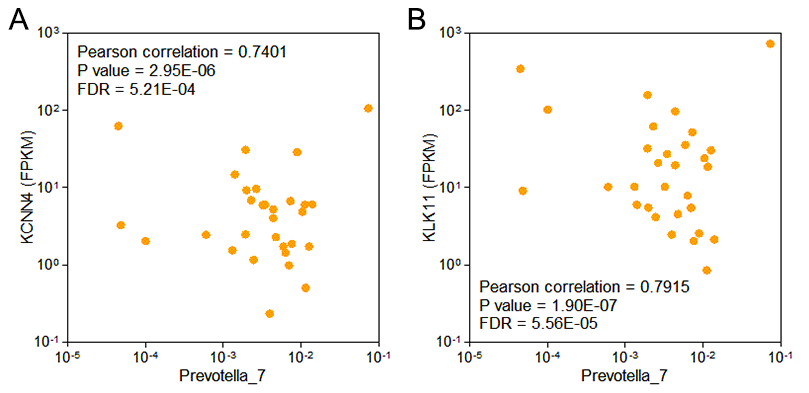


**Supplementary Figure 3.** Scatter plots of the *Prevotella*-gene pairs, *Prevotella*-KCNN4 (A) and *Prevotella*-KLK11 (B). Each point represents a patient.
